# The ERC1 scaffold protein implicated in cell motility drives the assembly of a liquid phase

**DOI:** 10.1038/s41598-019-49630-y

**Published:** 2019-09-19

**Authors:** Kristyna Sala, Agnese Corbetta, Claudia Minici, Diletta Tonoli, David H. Murray, Eugenia Cammarota, Lucrezia Ribolla, Martina Ramella, Riccardo Fesce, Davide Mazza, Massimo Degano, Ivan de Curtis

**Affiliations:** 1 Division of Neuroscience, San Raffaele Scientific Institute and Vita-Salute San Raffaele University, 20132 Milano, Italy; 2 Division of Immunology, Transplantation and Infectious Diseases, San Raffaele Scientific Institute, 20132 Milano, Italy; 3 Division of Cell and Developmental Biology, School of Life Sciences, University of Dundee, Dundee, United Kingdom; 4 Experimental Imaging Center, San Raffaele Scientific Institute, 20132 Milano, Italy; 5 Fondazione CEN, European Center for Nanomedicine, 20133 Milano, Italy; 6 Humanitas University, 20090 Pieve Emanuele, Italy

**Keywords:** Focal adhesion, Lamellipodia

## Abstract

Several cellular processes depend on networks of proteins assembled at specific sites near the plasma membrane. Scaffold proteins assemble these networks by recruiting relevant molecules. The scaffold protein ERC1/ELKS and its partners promote cell migration and invasion, and assemble into dynamic networks at the protruding edge of cells. Here by electron microscopy and single molecule analysis we identify ERC1 as an extended flexible dimer. We found that ERC1 scaffolds form cytoplasmic condensates with a behavior that is consistent with liquid phases that are modulated by a predicted disordered region of ERC1. These condensates specifically host partners of a network relevant to cell motility, including liprin-α1, which was unnecessary for the formation of condensates, but influenced their dynamic behavior. Phase separation at specific sites of the cell periphery may represent an elegant mechanism to control the assembly and turnover of dynamic scaffolds needed for the spatial localization and processing of molecules.

## Introduction

Migration through extracellular matrices requires protrusion at the cell front that is mediated by integrin adhesions^1^. The ERC/ELKS scaffold proteins and their partners liprin-α and LL5^2,3^ are regulators of a number of important cellular processes including cell migration and invasion^4,5^, the assembly of presynaptic active zones^6^ and cortical platforms linked to microtubules^7,8^. In migrating cells ERC, liprin-α and LL5 proteins form polarized plasma membrane-associated platforms (PMAPs)^9^ near the cell edge or near invadosomes^5,10^, to promote the turnover of adhesions/invadosomes and stimulate protrusion^11^. These scaffolds are distinct from exocytic/endocytic markers^5^. Supramolecular markers may harness liquid-liquid phase separation, giving rise to membrane-less organelles with specific functions within nucleus and cytoplasm^12^. Phase-separated systems may help the cell organizing molecules and reactions in space and time^12–15^. Interestingly, the propensity to undergo phase transition may be favored by intrinsically disordered protein regions (IDRs) characterized by lack of stable structure and increased flexibility^15,16^. The dynamic accumulation of ERC1/ELKS at sites of surface cell remodeling, and the lack of colocalization with membrane markers, led us to hypothesize that ERC1/ELKS may serve as a scaffold to assemble cytoplasmic condensates that include other components of a protein network relevant to cell motility and to other important cellular functions. Here we show that ERC1 can drive the formation of membrane-less condensates with liquid properties, and that ERC1-mediated condensates specifically host partners of a network relevant to cell motility, including liprin-α1. In this study we have used three types of cells: MDA-MB-231 human breast cancer cells and HT1080 human fibrosarcoma cells were used as examples of migratory cells accumulating ERC1 at the protruding leading edge, where ERC1 and associated proteins are known to play an important role in the regulation of cell edge dynamics^5,11^; while COS7 cells were used as a simple experimental system to characterize the formation and behavior of ERC1–positive condensates.

## Results and Discussion

### ERC1 induces cytoplasmic condensates with liquid-like properties

Endogenous and enhanced green fluorescent protein (GFP)–tagged ERC1 accumulated near protruding sites in migrating cells (Supplementary Fig. 1a,b; Movie 1). Endogenous ERC1 and its ligand proteins liprin-α1 and LL5 are expressed by the cell types used in this study: we have previously shown that the three proteins partially colocalize near the cell edge of migrating human breast cancer MDA-MB-231 cells^5^. We show here that they partially colocalize also at the front of migrating HT1080 human fibrosarcoma cells, a different type of motile tumor cell (Supplementary Fig. 1b). Moreover, in the cytoplasm of spread non-motile COS7 cells the three endogenous proteins accumulated near focal adhesions (Supplementary Fig. 1c).

**Figure 1:**
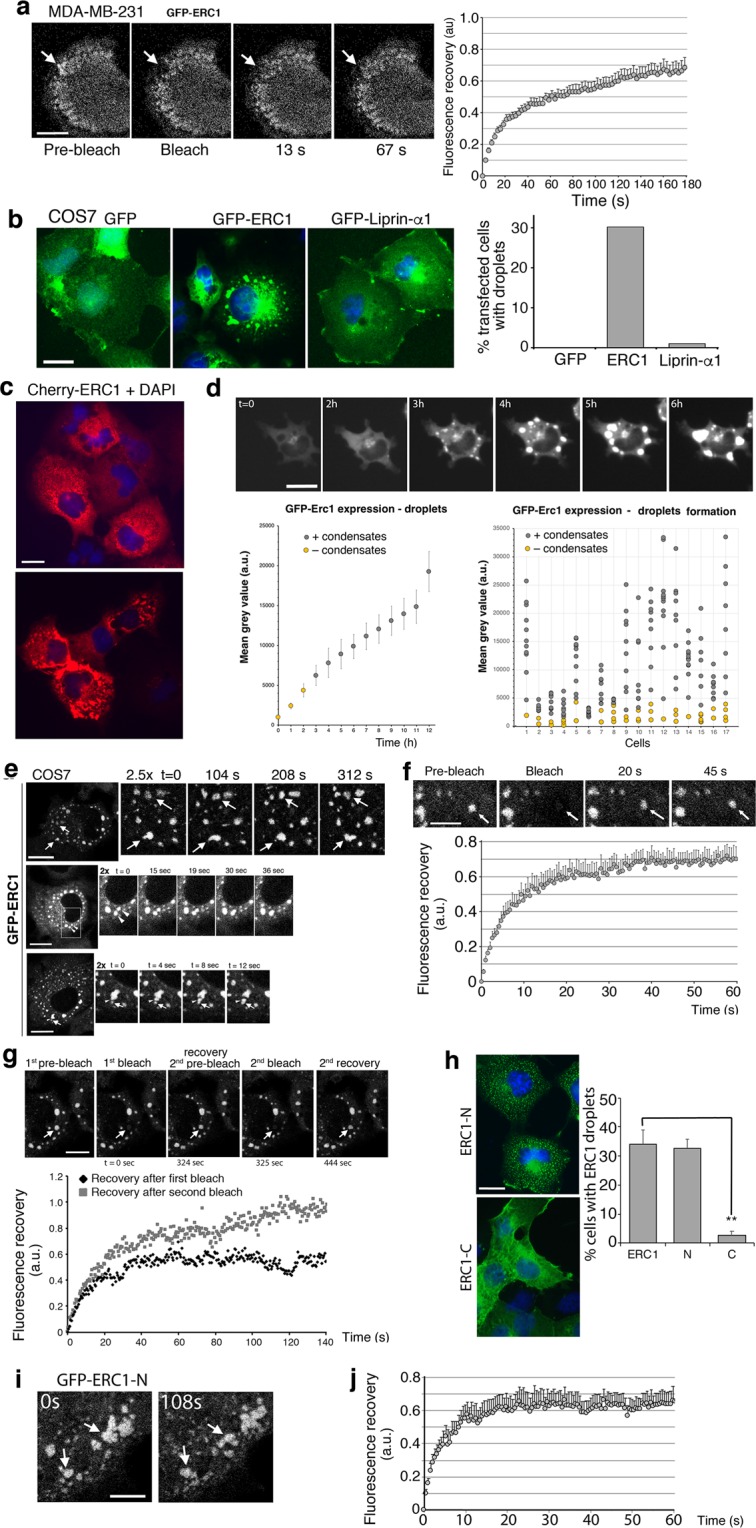
ERC1 induces cytoplasmic condensates with liquid-like properties. (**a**) Dynamic ERC1-positive PMAPs at the front of motile cells (confocal microscopy, basal membrane plane). Fluorescence recovery after photobleaching (See also Supplementary Movie 2) of GFP-ERC1 assemblies near the leading edge (arrow) in a migrating MDA-MB-231 cell. Scale bar, 10 µm. Right: fluorescence recovery, means ± SEM (n = 11). (**b**) Transfected COS7 cells analyzed with anti-tag Abs (green); blue is DAPI (widefield epifluorescence microscopy). Scale bar, 20 µm. Right: percentage of cells with condensates (n = 60–97 cells/condition). (**c**) COS7 cells plated on 2.5 μg/ml fibronectin–coated coverslips were transfected with mCherry-ERC1 and fixed after 24 h. Widefield epifluorescence microscopy of ERC1–positive condensates of different sizes were observed in several cells. Scale bar, 20 µm. (**d**) Top: GFP-ERC1 expression induces the formation of condensates in a concentration-dependent manner in the cytoplasm of COS7 cells. Scale bar, 25 µm. Bottom: in each transfected cell, the cytoplasmic fluorescence intensity (expressed as mean grey value, arbitrary units, a.u.) was measured every hour for 12 h, starting from the beginning of detectable GFP-ERC1 expression (n = 17 cells). The left graph shows the mean grey values ± SEM at each time point. In the right graph the GFP-ERC1 expression level (mean grey value) is plotted against the presence (grey dots) or absence (yellow dots) of detectable cytoplasmic condensates. (**e**–**g**) Confocal microscopy, central cytoplasmic plane. (**e**) Dynamic cytoplasmic GFP-ERC1 droplets with liquid–like behaviors. Top panels: left image, lower enlargement; arrows show examples of dynamic GFP-ERC1–positive droplets shown also in the enlargements on the right. Center: fusion between two GFP-ERC1–positive droplets in transfected cell (See also Supplementary Movie 4). Right: two-fold enlargements of the area of cytoplasm indicated by the white box in the cell shown on the left. Arrowheads indicate two droplets undergoing fusion. Bottom: splitting of GFP-ERC1–positive droplet (arrowheads) into two smaller droplets, followed by fusion of one of the new droplets (arrows) (See also Supplementary Movie 5). Right: 2-fold enlargements of the area indicated by arrows in the cell shown in the left panel. Bars, 20 µm. (**f**) FRAP on droplet (arrow) in the cytoplasm of COS7 cell transfected with GFP-ERC1 (See also Supplementary Movie 6). Scale bar, 8 µm. Bottom: fluorescence recovery (means ± SEM; n = 6 cells). (**g**) Frames from COS7 cell expressing GFP-ERC1: the same droplet (arrow) was bleached twice to evaluate fluorescence recovery. Bottom: quantification shows incomplete recovery after the first bleaching (black dots), which may be due either to the immobile fraction of molecules within ERC1 condensates, or to depletion of the GFP-tagged molecules in the cytosol. Full recovery of fluorescence after the second bleach (grey dots) confirmed that the immobile fraction was undetected after the second bleach. Scale bar, 20 µm. (**h**) Widefield epifluorescence microscopy showing GFP-ERC1–N cytoplasmic droplets (blue, DAPI). Percentage of transfected COS7 cells with droplets positive for different ERC1 constructs; means ± SEM; n = 3–4 experiments, 361–569 cells per condition; **p < 0.01. (**i**) Confocal microscopy, central cytoplasmic plane. Frames show liquid–like behavior (arrows) of cytoplasmic GFP-ERC1-N–positive droplets. Scale bar, 8 µm. (**j**) Means ± SEM of fluorescence recovery in GFP-ERC1-N droplets (n = 5).

The ERC1–positive PMAPs lack detectable membranous structures^5^ and lipid accumulation (Supplementary Fig. 1d). To assess the dynamics and exchange of scaffolds within this region, we analyzed these GFP-ERC1–positive PMAPs in MDA-MB-231 cells migrating on fibronectin by fluorescence recovery after photobleaching (FRAP). GFP-ERC1 fluorescence recovered with t_1/2_ = 19.9 ± 2 sec to 66 ± 6% of the initial fluorescence (Fig. 1a; Supplementary Movie 2), showing that ERC1 in PMAPs exchanged rapidly with cytosolic GFP-ERC1, as described for other intracellular liquid condensates^17,18^.

An increase in the local concentration of specific proteins may trigger phase transition^16^. We compared the behavior of PMAPs components ERC1 and liprin-α1 in cells. ERC1 accumulated into cytoplasmic condensates in 30% of transfected cells, while liprin-α1 remained diffuse in virtually all cells (Fig. 1b,c). At low levels of expression GFP-ERC1 displayed a homogeneous distribution throughout the cytoplasm and did not form condensates. ERC1 condensates appeared 2–4 h after transfection and increased in number and size with time (Supplementary Movie 3; Fig. 1d). When we plotted the presence of GFP-ERC1 condensates towards its level of expression, we observed that condensation occurs when ERC1 has reached a certain level of protein expression in the cytoplasm (Fig. 1d).

ERC1 condensates showed continuous shape changes with fusion and fission events (Fig. 1e; Supplementary Movies 4 and 5). Rapid recovery of fluorescence after full bleach of ERC1 condensates (t_1/2_ = 5.6 ± 0.56 s; 68% mobile fraction) revealed fast exchange of ERC1 between condensates and cytoplasm (Fig. 1f,g; Supplementary Movie 6).

Interestingly, the N-terminal region of ERC1 (ERC1-N), but not the C-terminal part (ERC1-C) formed cytoplasmic droplets with liquid-like behavior (Fig. 1h–i). Bleached ERC1-N droplets recovered 62 ± 7% of fluorescence with t_1/2_ = 3.0 ± 0.42 s (Fig. 1j), an exchange faster than observed for full length ERC1 (Fig. 1f).

### ERC1 forms extended homodimers

The structure within molecular scaffolds is in many cases poorly understood, and detailed information on ERC1 structure is missing. We have used predictions and electron microscopy on the purified ERC1 protein to address the mechanisms that may underlie the formation of condensates by this protein. Sequence–based structural prediction suggests that ERC1 adopts a dimeric coiled-coil structure^19^ (Supplementary Fig. 2). However, the 150 N-terminal residues and other shorter sequences throughout ERC1 were predicted to be IDRs^20^ with low probability to form coiled-coils. A number of features of the primary sequence of the first 147 residues of ERC1 support the structural disorder of the N-terminus of ERC1 (Supplementary Fig. 2). This region is characterized by low abundance of charged residues: total of Arg + Asp + Glu + Lys is 15% for ERC1–147 versus 36% for full length ERC1. ERC(1–147) is characterized by less negatively charged residues (<5% of Asp + Glu residues), resulting in a predicted total net charge at pH 7 of +8 (mean net charge per residue of +0.054; theoretical pI = 10.9), compared to the predicted total net charge at pH 7 of –27 for the full length ERC1 (mean net charge per residue of –0.024; theoretical pI = 5.7). As observed for disordered proteins^21^, this region includes 63% of disorder-promoting residues, and is especially enriched in disorder-promoter Gly, Pro and Ser residues (Supplementary Fig. 2). Moreover a low complexity region (LCR) was found by the SEG program (http://www.biology.wustl.edu/gcg/seg.html) corresponding to residues 13 to 51 of the predicted N-terminal IDR. LCRs represent a feature commonly found within disordered regions^16^.

**Figure 2:**
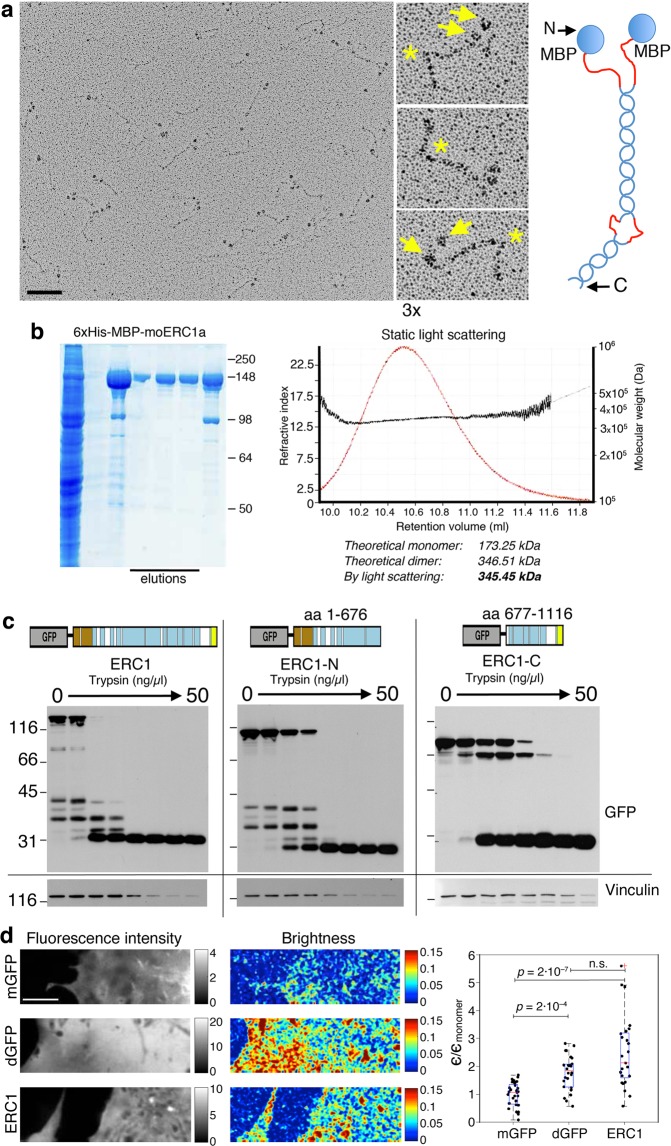
ERC1 forms homodimers that are sensitive to protease. See also Supplementary Fig. S3. (**a**) Rotary shadowing electron microscopy of purified MBP-ERC1. Globular portions (MBP) fused to the N-terminus of ERC1. Scale bar, 100 nm. Center: ERC1 dimers with two globular portions (arrows) and a bent often observed in the elongated portion (asterisks). Right: model of ERC1 parallel homodimers: in red predicted flexible segments. (**b**) Left: SDS-PAGE of affinity purified MBP-ERC1. Right: light scattering of purified MBP-ERC1 indicates its behavior as a dimer in solution. (**c**) Cell lysates incubated 5 min at 0 °C with trypsin and blotted with anti-GFP Ab. At the highest trypsin concentration only a 30 kDa band corresponding to GFP was detected. Blots from three different gels are shown, separated by the vertical lines. Moreover, the horizontal line separates each upper blot (incubated with anti-GFP Ab) from the corresponding blot below, that corresponds to the upper part of the same filters re-incubated with anti-vinculin Ab to reveal endogenous vinculin. (**d**) Fluorescent intensity and brightness map of cells expressing monomeric GFP (mGFP), dimeric GFP (dGFP) and GFP-ERC1 (ERC1). The mean brightness of dGFP and ERC1 is higher than the brightness of mGFP. The brightness map was smoothed with a Gaussian filter with standard deviation 1 pixel. Scale bar, 5 µm. Right: brightness fold change of cytoplasmic dGFP and ERC1 compared to mGFP. The median ERC brightness (ϵ_ERC1_⁄ϵ_mGFP_ = 2.1) is comparable with the dimer’s one (ϵ_dGFP_⁄ϵ_mGFP_ = 1.8); n = 28 (mGFP), 26 (dGFP), and 25 (GFP-ERC1); non-parametric one-way ANOVA.

We experimentally verified the low–resolution structure of ERC1 by rotary shadowing electron microscopy on recombinant MBP-tagged ERC1. This analysis revealed that ERC1 forms parallel homodimers of about 119 ± 18 nm (n = 29) (Fig. 2a). Dimerization was confirmed by static light scattering (Fig. 2b). The variable distance between the two globular portions in different dimers suggested that the MBP tag was linked to the flexible disordered N-terminus of ERC1. This first description of the general structure of ERC1 is coherent with an elongated protein with extensive coiled coils and flexible N-terminus.

In support of the extended/partially disordered structure predicted for ERC1 we used limited proteolysis^22^, since extended and/or disordered proteins are more sensitive to proteases than structured proteins^23^. Lysates from cells expressing GFP-tagged full length, N–terminal (ERC1-N, residues 1–676) or C–terminal polypeptides (ERC1-C, residues residues 677–1116) showed loss of full polypeptides and appearance of fragments at increasing trypsin concentrations (Fig. 2c; Supplementary Fig. 3), with a band the size of GFP (≈30 kDa) remaining as unique trypsin-resistant fragment detectable by immunoblotting with anti-GFP antibodies at the highest trypsin concentration tested. The same filters reprobed for vinculin showed lower sensitivity to trypsin of this structured protein.

To verify if ERC1 is a dimer in the cytoplasm of living cells, we harnessed the Numbers & Brightness (N&B) technique^24^. Comparison of fluorescent intensity maps showed that the average brightness (ϵ) of GFP-ERC1 was similar to that of dimeric GFP (dGFP), and higher than monomeric GFP (mGFP): the ratio ϵ_ERC_ ⁄ϵ_mGFP_ = 2.1, was similar to ϵ_dGFP_⁄ϵ_mGFP_ = 1.8 (Fig. 2d), indicating that cytosolic ERC1 was dimeric. The oligomerization state of ERC1 in droplets couldn’t be tested with N&B, as the conditions were out of the range for applicability of this technique.

### The N-terminal region of ERC1 influences the properties of cytoplasmic condensates

**Figure 3:**
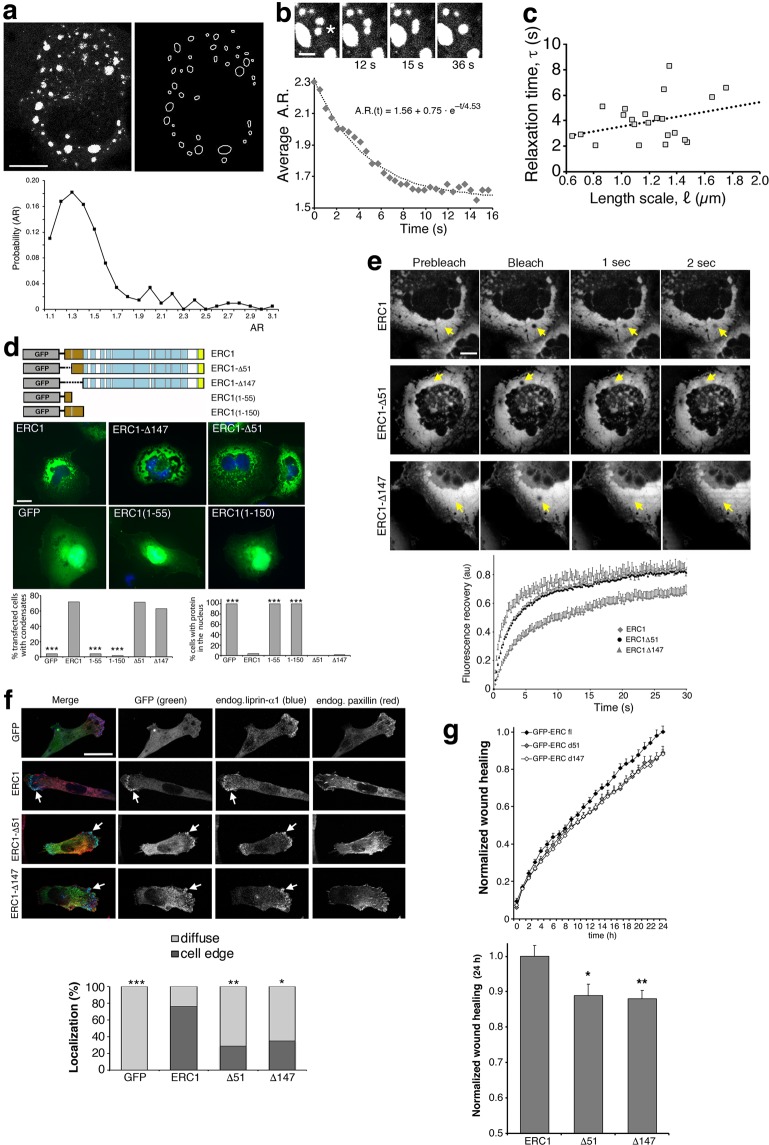
The N-terminal IDR of ERC1 is not required for the formation of cytoplasmic condensates. (**a**,**b**) Confocal microscopy, central cytoplasmic plane. (**a**) Top: fluorescent image and contour of cytoplasmic ERC1–positive droplets in COS7 cells. Scale bar, 20 µm. Bottom: distribution of A.R. from the analysis in fixed cells expressing GFP-ERC1 (n = 209 droplets; mean A.R. = 1.43 ± 0.026 SEM). (**b**) Fusion of ERC1 droplets in COS7 cells (asterisk). Scale bar, 5 µm. Bottom: dynamics of A.R. during fusion between pairs of droplets (n = 22). (**c**) Relaxation time versus length scale for fusions. Dotted line, linear regression (n = 22). The slope is an estimate of η/γ, inverse capillary velocity^25^. (**d**) Widefield epifluorescence microscopy of wildtype, ERC1-Δ51 and ERC1-Δ147 proteins form cytoplasmic condensates in COS7 cells. Scale bar, 20 µm. Bottom: percentage of transfected cells with cytoplasmic droplets (left) and with nuclear localization of the ERC1 constructs (right); n = 102–165 cells; ***p < 0.001 by the χ^2^ test vs GFP-ERC1. (**e**) Confocal microscopy, central cytoplasmic plane; FRAP after bleaching of 2 µm diameter spots (yellow arrows) on condensates in COS7 cells transfected with the indicated ERC1 constructs (frames from Supplementary Movies 7–9). Scale bars, 20 µm. Bottom: recovery of fluorescence after photobleaching: means ± SEM (n = 12–14 cells). (**f**) Confocal basal membrane plane of MDA-MB-231 cells expressing GFP constructs showing the transfected proteins and endogenous proteins liprin-α1 (blue) and paxillin (red). Scale bar, 20 µm. Graph below is the quantification of cells with GFP constructs accumulating at the cell edge. Significant differences were evaluated by the χ^2^ test versus GFP-ERC1 (n = 17–19 cells per condition). (**g**) Top: time course of the migration of MDA-MB-231 cells expressing GFP-tagged constructs into the wound. Bottom: migration at 24 h (complete wound closure); n = 32 fields from 4 experiments.

The dynamic behavior of fusing cytoplasmic ERC1 assemblies is consistent with their evaluation as liquid-like particles (Fig. 1e). We quantitatively analyzed the fusion events^25,26^ to gain insight into the biophysical properties of ERC1 condensates. The aspect ratio (A.R.) of smaller droplets was 1.43 ± 0.026 (Fig. 3a); deviation from round shape (A.R. = 1) was possibly due to constrains imposed by the crowded cellular environment, as indicated by the finding that larger condensates had dynamic irregular shapes (Supplementary Movie 3). Time course of fusion events leading to a larger droplet indicated that fusion occurred by exponential relaxation (relaxation time τ = 4.53 s) (Fig. 3b), as for coalescing liquids^26^. Plots of τ versus ℓ values (ℓ, length scale = size of the droplet) from several fusion events showed that the time constant of relaxation was correlated to the size of ERC1 assemblies (*R*^2^ = 0.29) (Fig. 3c), which is consistent with a liquid-like behavior. For liquid droplets, τ  = η/γ·ℓ (η, viscosity; γ, surface tension)^27^. Regression of τ over ℓ values gave an inverse capillary velocity η/γ  = 3.49 s.µm^-1^ (Fig. 3c). Similarly, the average of τ/ℓ ratios for each fusion event gave η/γ = 3.38 ± 0.39 s·µm^-1^. We considered the average value of η/γ  = 3.4 s·µm^-1^.

The surface tension (γ) of polymers undergoing phase separation scales as γ ≈ *k*_B_*T*/ξ^2^ (*k*_B_, Boltzmann constant; T, absolute temperature; ξ, length scale of the protein)^25,28^. Estimate for ERC1 dimers gave ξ^2^  = 628 nm^2^ (see Materials and Methods), with surface tension γ ≈ 6.8 μN·m^-1^. Based on the estimated inverse capillary velocity (η/γ = 3.4 s·µm^-1^), the apparent viscosity was estimated as η_APP_ = η/γ · γ = 23 Pa·s for ERC1 condensates, about 10^4^ times larger than cytosol^29^, and in the range of viscosities of other macromolecular liquid assemblies^25,30^.

Phase separation of proteins may be driven by disordered regions^16^, and has been observed also for proteins with predicted coiled-coil regions implicated in the assembly of the centrosomes^31^ and synapses^32^. We tested if the disordered N-terminus of ERC1 (Supplementary Fig. 2) was required for the formation of the condensates. The ERC1 proteins lacking either the low complexity region (ERC1-Δ51) or the full predicted N-terminal IDR (ERC1-Δ147) could form condensates like the full length ERC1, indicating that the predicted disordered N-terminal region is not necessary for the assembly of ERC1 condensates. Moreover the amino-terminal ERC1(1–55) and ERC1(1–150) fragments were both cytosolic, and therefore not sufficient to induce the formation of condensates (Fig. 3d). ERC1-Δ147 showed a non-significant trend to form larger condensates compared to full length ERC1 (Supplementary Fig. 4). Thus the predicted disordered N-terminal region was neither sufficient nor necessary for the assembly of ERC1 scaffolds that behave as liquid–like compartments in living cells.

In cells overexpressing either the full length ERC1 protein, or the ERC1-Δ147 and ERC1-Δ51 mutants, assemblies often grew to very large cytoplasmic condensates (Supplementary Fig. 5; Movie 3). Spot bleaching of small areas within these large condensates was followed by fast recovery (t_1/2_ = 4.4 ± 0.41 s; 69% ± 4% mobile fraction) (Fig. 3e; Supplementary Movie 7), as after full bleach of ERC1 condensates (Supplementary Movie 6). This indicated that in contrast to other membrane-less condensates that mature from liquid to more solid assemblies, the liquid properties of ERC1 condensates did not change when increasing in size over time. Interestingly, both ERC1-Δ51 and ERC1-Δ147 increased the percentage of fluorescence recovery and the speed of recovery compared to full length ERC1. The mobile fraction was 69% ± 4% for full length, 83 ± 2% for ERC1-Δ51 (*p* = 0.011), and 83 ± 4% for ERC1-Δ147 (*p* = 0.025); t_1/2_ was 4.4 ± 0.41 s for full length, 2.6 ± 0.2 s for ERC1-Δ51 (*p* = 0.001), and 1.8 ± 0.3 s for ERC1-Δ147 (*p* < 0.001) (Fig. 3e; Movies 7–9). Progressive deletion of the N-terminal disordered region led to condensates with enhanced liquid-like behavior.

We next evaluated the effects of the N-terminal deletions of ERC1 in migrating cells. Cell motility is supported by ERC1 and the associated proteins liprin-α1 and LL5^5,11^, which accumulate near the leading edge of the migrating cells (Fig. 1a; Supplementary Fig. 1a,b). In MDA-MB-231 cells migrating on fibronectin, the ERC1 constructs lacking the N-terminal IDR could still partially colocalize with endogenous liprin-α1 at condensates near the leading edge, but less efficiently than the full length ERC1 (Fig. 3f). These findings suggest that although the N-terminal IDR is not required for the formation of the condensates, it may be relevant to stabilize the condensates that are needed to recruit the interacting partners at the protruding edge of the migrating cells. In this direction, we observed that the truncated form GFP-ERC1-Δ147 lacking the predicted N-terminal IDR had a prevalent diffuse distribution in most transfected MDA-MB-231 cells migrating on fibronectin, compared to the full length GFP-ERC1 protein that localized at the front of most migrating cells (Fig. 3f). Our previous findings showed that the localization of endogenous liprin-α1 at the edge of migrating cells was only mildly affected by the lack of ERC1^5^. On the other hand endogenous liprin-α1 was required for the recruitment of endogenous ERC1 at the cell edge^5^. In this direction, here endogenous liprin-α1 could still localize at the front of polarized cells expressing the GFP-ERC1-Δ147 mutant, where it partially colocalized with the ERC1 mutant (Fig. 3f). The fact that GFP-ERC1-Δ147 was largely diffuse may be a consequence of the decreased stability (i.e. increased liquid-like properties) of the ERC1-Δ147 condensates forming at the edge of the migrating cells.

Interestingly, the migration of MDA-MB-231 cells was moderately but significantly inhibited by the expression of either deletion mutant compared to full length ERC1 (Fig. 3g). These results suggest that the changes detected in the liquid properties of the ERC1 condensates formed by IDR–deleted proteins (Fig. 3e) may underlie the observed effects on the subcellular distribution of the condensates in migrating cells, and the moderate effects observed in the migration of the cells that express these mutants. On the other hand we can not exclude the possibility that these changes are due to loss of specific, so far unknown protein interactions with the amino-terminal end region of ERC1.

### ERC1–induced condensates recruit PMAP components and are affected by liprin-α1

**Figure 4:**
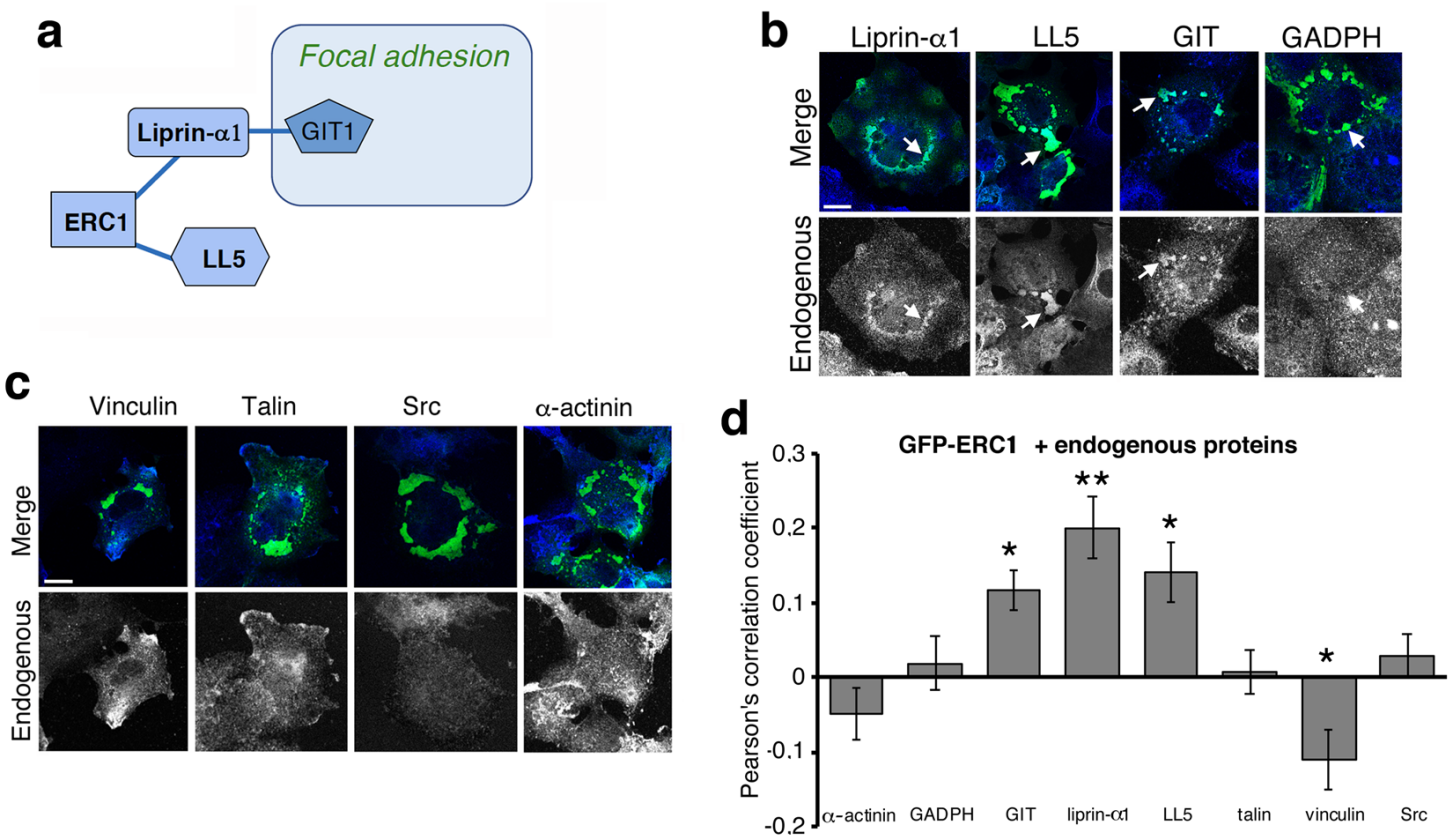
ERC1–induced condensates recruit PMAP components. (**a**) Network of proteins recruited to ERC1-positive droplets. Blue lines indicate direct interactions between proteins, as from published work. (**b**,**c**) Confocal central cytoplasmic planes: (**b**) COS7 cells transfected with GFP-ERC1 (green), immunostained for endogenous proteins; arrows show examples of endogenous proteins (blue) at ERC1 droplets (green) (scale bar, 20 µm); (**c**) distribution of endogenous proteins (bottom) in COS7 cells with ERC1–positive droplets; no clear localization of endogenous vinculin, talin, Src and α-actinin (blue) at ERC1-positive droplets (green). Scale bar, 20 µm. (**d**) Pearson’s correlation coefficient for the colocalization of endogenous proteins at ERC1 droplets (n = 19–35); *p < 0.05, **p < 0.01 (t-test, endogenous GAPDH as control).

Liprin-α1, LL5 and GIT proteins are part of a network regulating protrusion and focal adhesions (Fig. 4a)^5,33^. These proteins were specifically recruited to ERC1 condensates (Fig. 4b), while a control cytosolic enzyme (GADPH, glyceraldehyde 3-phosphate dehydrogenase) and other focal adhesion proteins such as α-actinin, talin, vinculin and Src were excluded (Fig. 4c). These observations were quantified as percentage of ERC1–positive condensates including each of the different endogenous proteins (Fig. 4d, Supplementary Fig. 6a)). These results show that ERC1 condensates selectively recruit client proteins that regulate cell motility.

**Figure 5:**
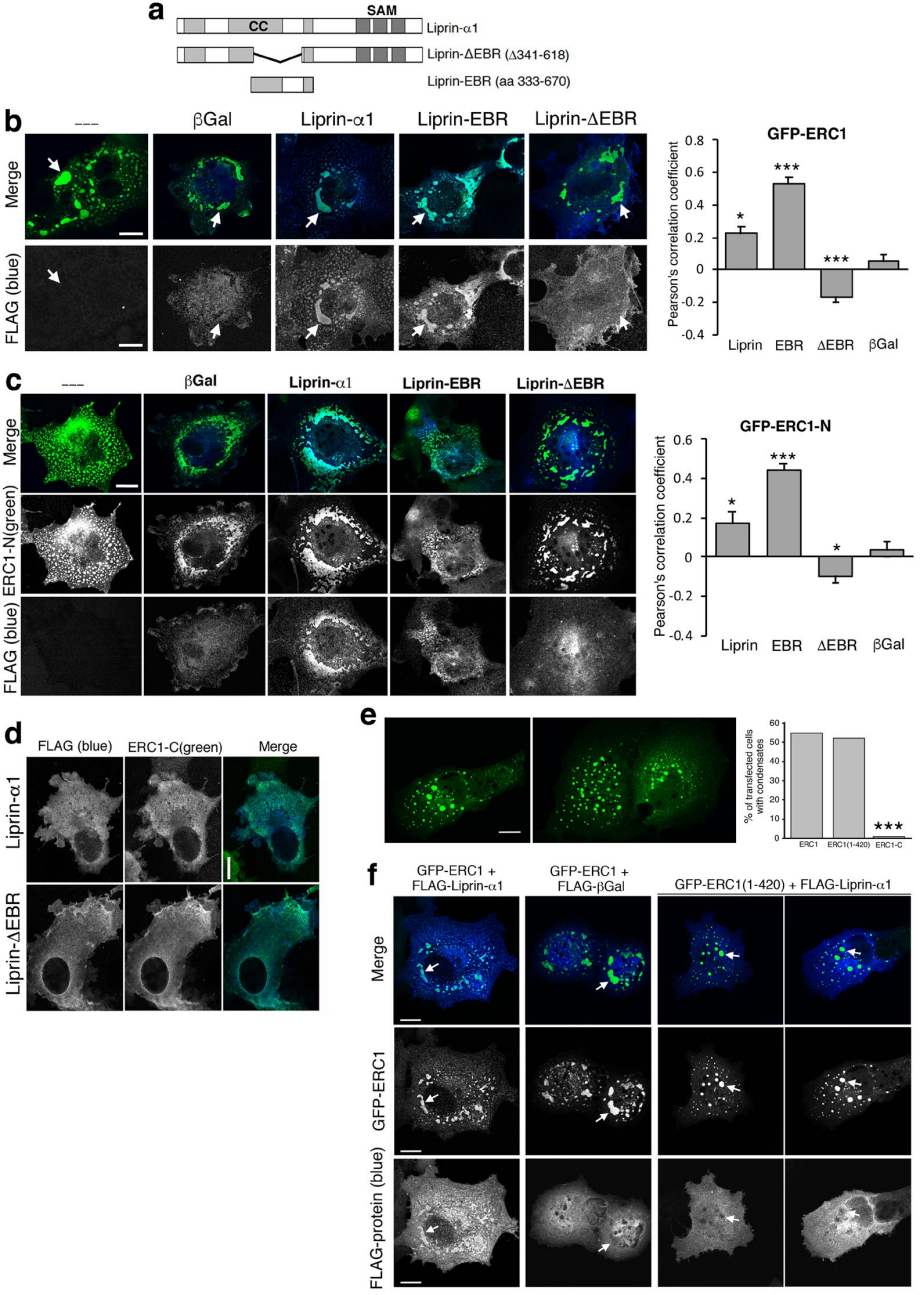
Liprin-α1 recruitment affects the properties of ERC1–induced condensates. (**a**) Flag-liprin-α1 constructs used in the study. (**b**–**d**) Confocal central cytoplasmic planes. (**b**) Cells co-expressing GFP-ERC1 (green) with FLAG-tagged constructs (blue). Scale bars, 20 µm. Right: Pearson’s correlation coefficient for the colocalization of liprin-α1 constructs at ERC1 droplets (n = 20–21); *p < 0.05; ***p < 0.001 (t-test, β-galactosidase as control). (**c**,**d**) The ERC1–binding region of liprin-α1 is required for the localization of liprin-α1 at ERC1-N droplets. Confocal microscopy of COS7 cells revealing GFP-ERC1-N (**c**), or GFP-ERC1-C (**d**) (in green), and the indicated FLAG-tagged constructs (in blue). Scale bars, 20 µm. Right panel in (**c**): Pearson’s correlation coefficient for the colocalization of liprin-α1 constructs at ERC1-N droplets (n = 19–26); *p < 0.05; ***p < 0.001 (t-test, β-galactosidase as control). (**e**) Left: confocal microscopy of GFP-ERC1(1–420) condensates in COS7 cells. Right: quantification of the percentage of transfected cells with condensates (n = 131–147 transfected cells per experimental condition; ***p < 0.001, χ^2^ test). No significant difference observed between cells transfected with either ERC1(1–420) or full length ERC1. (**f**) Confocal microscopy of COS7 cells co-expressing the indicated FLAG-tagged proteins with either GFP-ERC1 (two left columns) or GFP-ERC1(1–420) (two right columns). Arrows point to the same condensates in merge and single channels.

The interaction of ERC/ELKS with liprin-α is relevant to a number of important cellular processes^2,6,11,34,35^. ERC proteins interact with the central ERC–binding region (EBR) of liprin-α^2^ (Fig. 5a). Deletion of the EBR prevented the recruitment of liprin-ΔEBR to both ERC1– and ERC1-N–induced condensates, while the EBR fragment was efficiently recruited to both ERC1 and ERC1-N condensates (Fig. 5b,c, Supplementary Fig. 6b). Liprin-α1 remained diffuse in cells co-expressing ERC1-C (Fig. 5d). These data show that the EBR is necessary and sufficient for the recruitment of liprin-α1 to ERC1 condensates.

We then tested the effects of endogenous liprin-α1 and LL5 proteins on the formation of condensates. Silencing of liprin-α1 did not affect droplets formation (Supplementary Fig. 7A); also silencing of LL5 did not significantly influence the formation of ERC1 condensates (Supplementary Fig. 7b).

We have previously shown that endogenous liprin-α1 is required for the subcellular concentration of ERC1 at PMAPs forming near the leading edge of migrating MDA-MB-231 tumor cells^5^. We hypothesize that liprin-α1 is required for the local concentration of the endogenous/low expressed ERC1 protein at specific sites of the cells, thus allowing the condensation of the protein by phase separation when and where it is required. According to this hypothesis, if the cells express higher levels of the protein, the condensates may form by overcoming a threshold concentration that allows phase separation to occur independently of specific localized signals. The finding that high levels of expression of ERC1 allow the formation of condensates in the cytoplasm even after depletion of endogenous liprin-α1, shows that liprin-α1 is not necessary for the process of ERC1-induced phase separation *per se*. This is confirmed also by the finding that the GFP-tagged amino-terminal construct ERC1(1–420) lacking a large part of the minimal region required for binding to liprin-α proteins (corresponding to amino acid residues 126–567 of ERC1^2^) is still able to efficiently induce the formation of condensates (Fig. 5e). The full length FLAG-Liprin-α1 protein recruited at condensates induced by full length GFP-ERC1 was indeed excluded by the condensates induced by GFP-ERC1(1–420) (Fig. 5f).

**Figure 6:**
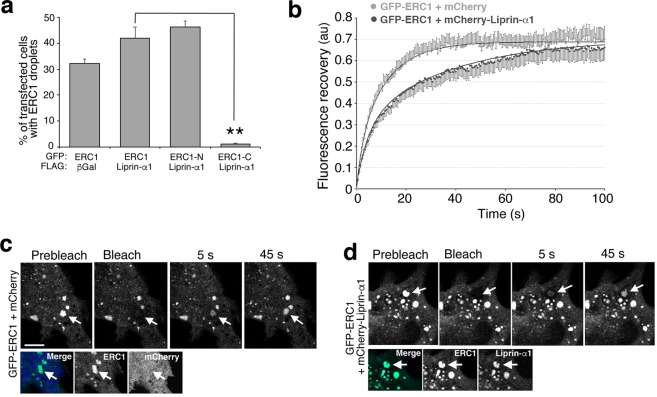
Liprin-α1 recruitment affects the properties of ERC1–induced condensates. (**a**) Percentage of cotransfected cells (GFP-tagged ERC1 constructs and FLAG-liprin-α1 or FLAG-βgalactosidase constructs) with droplets; **p < 0.01; n = 3–4, 310–539 transfected cells per condition analyzed. (**b**) Recovery of ERC1 fluorescence in cells co-expressing GFP-ERC1 and mCherry (light grey; n = 10), or GFP-ERC1 and mCherry-Liprin-α1 (dark grey; n = 14 cells). (**c**,**d**) Confocal central cytoplasmic plane. FRAP on droplets (arrows) in cells co-expressing GFP-ERC1 with either mCherry (**c**) or mCherry-Liprin-α1 (**d**). Scale bars, 10 µm.

The recruitment of FLAG-Liprin-α1 at ERC1 condensates did not significantly affect their formation (Fig. 6a). Liprin-α1 slowed the recovery of fluorescence after the bleaching of droplets co-expressing ERC1 and liprin-α1 (t1/2 = 9.1 ± 1.0 s) compared to control ERC1 droplets (t1/2 = 5.3 ± 0.4 s; *p* = 0.0024) (Fig. 6b–d), without affecting the ERC1 mobile fraction (65 ± 5.4% for ERC1/mCherry-Liprin-α1; 67 ± 5% for ERC1/mCherry; *p* = 0.81). Thus liprin-α1 slows down the exchange of ERC1 between the condensates and the surrounding cytosol. This effect may be relevant to the finding that liprin-α1 is required for the recruitment of ERC1 at the protrusive edge of migrating cells^11^.

## Conclusions

Phase separation near specific sites of the cell periphery presents an elegant cellular mechanism to control the spatial localization and processing of molecules. These findings indicate that ERC1 scaffolds may behave as cytoplasmic liquid phases that host binding partners belonging to a protein network that has been implicated in the regulation of cell motility^5,11^. This may provide a way to promote the turnover of molecular components and to recruit enzymes such as kinases and/or phosphatases needed to sustain the protrusive activity of motile cells, possibly by modulating the effects and activity of specific components. Our evaluation of ERC1/ELKS-mediated scaffolding as condensates provides an intellectual framework for future analysis of other large molecular networks including this protein, such as the active zone of synapses^6^.

## Materials and Methods

### Plasmids

Deletion mutants for murine ERC1/ELKSε) were obtained by PCR from plasmid GFP-ERC1a. The following primers were used: 5′-GGAATTCCATGAAAACCCTTTCAATGGAG-3′ and 5′-GGAATTCTCAGGAGGACTCTTCCAG-3′ for GFP-ERC1-Δ51; 5′-GGAATTCCATGGAT AACACCATCATGGATCTG-3′ and 5′-GGAATTCTCAGGAGGACTCTTCCAG-3′ for GFP-ERC1-Δ147; 5′-GGAATTCCATGTATGGAAGTGCTCGATC-3′ and 5′-GGAATTCTTATGA AAGGGTTTTCCCACTG-3′ for GFP-ERC1(1–55); 5′-GGAATTCCATGTATGGAAGTGC TCGATC-3′ and 5′-GGAATTCTTAGGTGTTATCTCTTGCCTGACG-3′ for GFP-ERC1(1–150); 5′-GGAATTCCATGTATGGAAGTGCTCGATC-3′ and 5′-GGAATTCTCACTCCCGCT CCTCACTAC-3′ for GFP-ERC1(1–420). Plasmids for GFP-Liprin-α1, FLAG-Liprin-α1, FLAG-Liprin-ΔEBR, FLAG-Liprin-EBR, FLAG-βGal, mCherry-ERC1 and mCherry-Liprin-α1 were described previously^5,10,11,36,37^. pEGFP-C1 and mCherry-C1 are commercially available (Clontech Laboratories, MountainView, CA, USA). The pEGFP-ERC1 plasmid (coding for the murine ELKSε/ERC1a isoform) was from Dr. Y. Takai^38^. The pEGFP-ERC1-N construct coding for the GFP-tagged N-terminal fragment of ERC1 (residues 1–676) was obtained by ligation of pEGFP-ERC1 digested with EcoR V and Sma I. The pEGFP-ERC1-C plasmid coding for the GFP-tagged C-terminal fragment of ERC1 (residues 677–1116) was obtained by digestion with Hind III and EcoR V, blunting and ligation of pEGFP-ERC1. To obtain His-tagged ERC1-N, the ERC1-N fragment was obtained by PCR with site-directed mutagenesis on GFP-ERC1-N to introduce a STOP codon; the resulting ERC1-N fragment was subcloned in pET28b. To obtain the pET28b-ERC1-C plasmid, the ERC1-C fragment was excised from pEGFP-ERC1-C by digestion with Xho I, blunting and digestion with BamH I. The ERC1-C fragment was inserted into the pET28b vector digested with Nhe I and BamH I and blunted.

### SiRNAs

Liprin-α1, ERC1 and LL5α and β were silenced by transfecting validated siRNAs as described^11^. Targeting sequence for siRNA were 5′-CACGAGGTTGGTCATGAAAGA-3′ for liprin-α1 (Qiagen, Hilden, Germany); 5′-CCAACAGTACGGGAGGGAG-3′ for ERC1, 5′- CCATCAGCCTGAGTGAATA-3′ for LL5α, 5′-GGAGATTTTGGATCATCTA-3′ for LL5β, and 5′-CATCACGTACGCGGAATAC-3′ for control luciferase (Life-Technologies, Paisley, UK).

### Antibodies

Chicken pAb anti-GFP, mAb anti-GAPDH, and mAb anti-N-terminal ERC1a (ELKS-30) against residues 21–40 of mouse/human ERC1a (Abcam, Cambridge, UK); rabbit pAb anti-GFP (Thermo Fisher Scientific, Paisley, UK); rabbit pAbs anti-FLAG and anti-LL5β, mAbs anti-α-actinin, anti-tubulin, and anti-talin (Sigma-Aldrich); mAb anti-vinculin (Upstate, Lake Placid, NY, USA); rabbit pAb against the C-terminus of liprin-α1 (amino acids 818–1202)^36^ pAb rabbit anti-liprin-α1 (Protein Tech, Chicago, IL, USA); mAbs anti-paxillin and anti-GIT proteins (BD Bioscience, San Jose, CA, USA); mAb anti-LL5 (cl. IH12) from J. Sanes^39^; mAb anti Src cl. 327 from S. Courtneidge^40^. Alexa-Fluor–conjugated secondary antibodies (Thermo Fisher Scientific, Paisley, UK); horseradish peroxidase (HRP)-conjugated secondary antibodies (Amersham Bioscience, Little Chalfont, UK). Lipophilic dye Nile Red (Sigma Aldrich, St. Louis, MO, USA).

### Cell culture and transfection

COS7 cells were cultured in DMEM with 10% fetal clone III (Hyclone), 100 U/ml penicillin, 100 μg/ml streptomycin, 20 mM glutamine. MDA-MB-231 cells were grown in DMEM/F12 1:1 with 10% fetal bovine serum (FBS). HT1080 human fibrosarcoma cells were cultured in EMEM with 10% FBS. Cells on plastic or on round 13–24 mm diameter glass coverslips were transfected with Lipofectamine-2000® (Thermo Fisher Scientific, Paisley, UK) and the indicated siRNA (50 nM) and/or plasmid (1–10 μg of DNA) for biochemistry or microscopy. Cells transfected only with plasmids were processed 24–30 hours after transfection. Cells transfected with siRNAs (alone or in combination with plasmids) were processed 48 hours after transfection. All siRNAs efficiently down-regulated the endogenous proteins.

### Live cell imaging and FRAP

For live cell imaging COS7 or MDA-MB-231 cells were plated at low density on 35 mm diameter glass bottom dishes (MatTek, Corporation, Ashland, MA) or on 24 mm diameter glass coverslips coated with fibronectin (1.5–2.5 μg/ml for 1 h at 37 °C) (BD, Biosciences, San Jose, CA, USA), and transfected with the indicated plasmids. Cells were recorded 24–48 h after transfection. Live cell imaging experiments were performed on an inverted laser scanning confocal microscope Leica TCS SP8 SMD-FLIM, equipped with HC PLAPO CS2 63x lens (NA 1.4), adaptive focus control and Oko-Lab stage incubator. To follow the formation of ERC1 droplets, cells were imaged for 1 day (one frame every 5 min) with a Live-Cell Imaging System equipped with 20x lens (Essen BioScience, Ann Arbor, MI). During image acquisition, cells were maintained in phenol red-free DMEM medium (Thermo Fischer Scientific, Paisley, UK) with 10% FBS, at 37 °C and 5% CO_2_. Data were pooled from 2–5 independent experiments.

For FRAP experiments, cells plated on 3.5 cm diameter glass-bottom MatTek dishes (MatTek Corporation, Ashland, MA) coated for 1 h at 37 °C with 2.5 μg/ml fibronectin were transfected for 24 h and imaged at a Leica TCS SP8 SMD FLIM confocal microscope equipped with HC PLAPO CS2 63x lens (NA 1.4), adaptive focus control and Oko-Lab stage incubator (T, CO_2_), and FRAP module with LasX software (Leica). Before imaging cells were moved to imaging medium without phenol red. For full droplet bleaching FRAP experiments, a 3–6 μm diameter circular region of interest (ROI) was bleached, with 70% laser power at 489 nm (white laser line), 10 iterations every 0.52 sec. After bleaching, images were taken every 0.52 sec for 2.5–5 min to monitor fluorescence recovery. The recovery was measured with ImageJ by calculating the fluorescent intensity at each time point as follows: R = [F(t)-F(0)]/[F(pre-bleach)-F(0)], where F(t) is the intensity of fluorescence at time t: F(t) = [F(ROI(t))-F(bcg(t))]/[F(ctrl(t))-F(bcg(t))], where “bcg” stands for background fluorescence outside the cell, and “ctrl” stands for control area in the cytosol for full droplet bleaching^41^.

For FRAP analysis after spot bleaching, a 2 μm diameter ROI was bleached with 70% laser power at 488 nm (argon laser line) with 1 iteration for 0.2 sec. After bleaching, images were taken every 0.2 sec for 2 min to monitor fluorescence recovery, which was calculated as described in the previous paragraph; for spot bleaching, “ctrl” stands for a nearby ERC1-positive condensate area.

### Immunofluorescence and image analysis

Transfected and non-transfected cells plated on 2.5 μg/ml fibronectin-coated glass coverslips were processed for immunofluorescence as described^37^. Briefly, cells were fixed for 10 min with 3% paraformaldehyde at room temperature, permeabilized with 0.1% saponin or Triton-X100 in PBS, incubated with primary Abs, washed, incubated with secondary Abs, and mounted with ProLong Gold antifade mounting solution (Thermo Fisher Scientific). Cells transfected only with GFP constructs were observed after fixation without enhancing the GFP signal with anti-GFP Abs. Cells were observed with epifluorescence microscopes: Zeiss AxioImager M2m equipped with AxioCam color CCD camera, with Plan-Neofluar 40x lens (NA 0.75) and Plan-Apochromat 63x lens (NA 1.4); with Zeiss Axiovert 135 TV with Hamamatsu CCD Orca II camera and Plan-Apochromat 63x lens (NA 1.4). Confocal images were acquired at a Perkin Elmer UltraVIEW spinning disk confocal microscope with EM-CCD camera and Plan-Apochromat 63x lens (NA 1.4); or at a Leica TCS SP8 SMD FLIM laser scanning confocal microscope equipped with HC PLAPO CS2 63x lens (NA 1.4). For quantitative analysis of cells forming droplets, transfected cells were randomly imaged at a wide field microscope. For quantification of droplets positive for endogenous proteins, confocal images were visually analyzed. For quantification, 2–4 independent experiments were used.

The formation of condensates was followed in COS7 cells plated for one day on fibronectin–coated dishes (2.5 μg/ml for 1 h at 37 °C), and then transfected to express GFP-ERC1. Right after transfection, the cells were imaged for 1 day with Live-Cell Imaging System equipped with 20x lens (Essen BioScience, Ann Arbor, MI) to follow the formation of cytoplasmic GFP-ERC1 droplets. For the graphs shown in Fig. 1d, the fluorescence intensity in each transfected cell was measured every hour for 12 h, starting from the beginning of detectable GFP-ERC1 expression, by using the Image J software.

### Bioinformatics

Amino acid sequences of ERC1a were obtained from UniProt (http://www.uniprot.org/). Data on their amino acid composition were extracted using the ProtParam tool (http://web.expasy.org/protparam/). For the analysis of primary sequences, we considered order-promoting (Asn, Cys, Ile, Leu, Phe, Trp, Tyr, Val) and disorder-promoting amino acids (Ala, Arg, Gln, Glu, Gly, Lys, Pro and Ser) as defined^21^. Data for globular proteins were extracted from published studies (42). Net charge at pH 7 was calculated as the difference between positively (Arg and Lys) and negatively charged (Glu and Asp) residues. The mean net charge was obtained by dividing the net charge values by the number of total amino acids in the sequence. Intrinsically disordered regions were identified utilizing the DisEMBL program (http://dis.embl.de/cgiDict.py) for intrinsic protein disorder prediction^20^.

### Characterization of the biophysical properties of ERC1–positive droplets in cells

#### Analysis of fusion events

Fusion events were analyzed in time on movies of transfected cells acquired for 5 m at a confocal microscope (TCS SP8 SMD-FLIM Leica equipped with HC PLAPO CS2 63x lens (NA 1.4), with adaptive focus control and Oko-Lab stage incubator). The aspect ratio (A.R.) of droplets was determined by ImageJ software, by fitting an ellipse to the shape of two fusing droplets and calculating A.R. = *ℓ*long/*ℓ*short (ratio between the longer [*ℓ*long] and the shorter [*ℓ*short] diameter of fusing droplets)^25^. A.R. were measured from time lapses and plotted as a function of time, and typically relaxed toward a steady value. For each fusion event between two droplets A.R. was measured at ${\rm{\delta }}t\,=\,0.52$ s intervals and its decline was fitted according to the exponential curve: ${\rm{A}}.{\rm{R}}.({\rm{t}})={\rm{A}}.{\rm{R}}{.}_{\infty }+({\rm{A}}.{\rm{R}}{.}_{0}-{\rm{A}}.{\rm{R}}{.}_{\infty }\,)\cdot \exp (\,-\,{\rm{t}}/{\rm{\tau }})$; ${\rm{\tau }}$ (the timescale or relaxation time for fusion) and ${\rm{A}}.{\rm{R}}{.}_{\infty }$ were estimated from the autoregressive relation:

$${\rm{A}}.{\rm{R}}.({\rm{t}}+{\rm{\delta }}t)={\rm{A}}.{\rm{R}}{.}_{{\rm{n}}+1}={\rm{a}}\cdot {\rm{A}}.{\rm{R}}{.}_{{\rm{n}}}+{\rm{c}}$$, where $${\rm{a}}=\exp (\,-\,{\rm{\delta }}t/{\rm{\tau }})$$ and $${\rm{c}}={\rm{A}}.{\rm{R}}{.}_{\infty }/(1-{\rm{a}})$$.

In particular, $$|\begin{array}{c}a\\ c\end{array}|=|\begin{array}{cc}{\rm{A}}.{\rm{R}}{.}_{0} & 1\\ {\rm{A}}.{\rm{R}}{.}_{1} & 1\\ \mathrm{..} & \mathrm{..}\\ {\rm{A}}.{\rm{R}}{.}_{n-1} & 1\end{array}|\backslash |\begin{array}{c}{\rm{A}}.{\rm{R}}{.}_{1}\\ {\rm{A}}.{\rm{R}}{.}_{2}\\ \mathrm{..}\\ {\rm{A}}.{\rm{R}}{.}_{{\rm{n}}}\end{array}|$$; ${\rm{\tau }}=-\,{\rm{\delta }}t/\,\log \,{\rm{a}}$ and ${\rm{A}}.{\rm{R}}{.}_{\infty }=(1-{\rm{a}})\cdot {\rm{c}}$.

The relaxation time τ for fusion is expected to be directly proportional to the length scale *ℓ* of the droplets according to the relation: τ ≈ (η/γ) · *ℓ*^25^. The ratio between the viscosity of the droplet (η) and its surface tension (γ) is called inverse capillary velocity: η/γ ≈ τ/*ℓ*. To measure the inverse capillary velocity η/γ, the dynamics of fusion between pairs of ERC1–positive droplets was analyzed by measuring the A.R. as a function of time for droplets with an area of up to 10 µm^2^ at the time of contact before fusion. The inverse capillary velocity of the system was estimated: (1) from the average value of the ratio of ${\rm{\tau }}$ to length scale (*ℓ*) for each droplet pair (i.e. the diameter of the droplet pair at the beginning of fusion); *ℓ* = [(*ℓ*_long_ (t = 0) - *ℓ*_short_ (t = 0)) · *ℓ*_short_ (t = 0)]^1/2^ ^25^; (2) alternatively, it was estimated from the regression of the set of $\tau$ values on the corresponding length scales. The same fit was performed on the grouped measurements from the fusion events and a further estimate of inverse capillary velocity was obtained from the resulting $\tau$ value and the average length scale.

The determination of the length scale of ERC1 dimers (ξ) was as follows. Based on the structural features of the ERC1 dimers revealed by rotary shadowing electron microscopy, we can approximate the ERC1 dimer, made of two 128 kDa monomers (each made of 1116 residues), to a cylinder with length *l* ≈ 100 nm and with a radius r ≈ 1 nm^42^. This gives a surface area ≈ 2·π·r × *l* = 628 nm^2^. The length scale (ξ) can be defined as the square root of the surface area of the protein molecule; therefore ξ^2^ = 628 nm^2^.

### Numbers & brightness

The plasmids coding for the monomeric mEGFP-GPI and the dimeric mEGFPmEGFP-GPI constructs were a generous gift of Dr. Andrea Orsi and Eelco van Anken (San Raffaele Scientific Institute, Milano). From these plasmids we obtained by site-directed mutagenesis the plasmids coding for the monomeric mEGFP and dimeric mEGFPmEGFP respectively, introducing a stop codon between the EGFP and the sequence signal coding for the capability of GPI lipid anchoring. COS7 cells plated on 3.5 cm diameter glass-bottom MatTek dishes coated for 1 h at 37 °C with 2.5 μg/ml fibronectin were transfected for 24 h. Before imaging cells were moved to imaging medium without phenol red.

#### Image acquisition

For each condition, time series of 300 images were acquired with a Leica SP8 confocal microscope with the following settings: frame size 512 × 64 pixels, speed 400 Hz, pinhole size 1 AU. These settings correspond to a pixel size of 120.37 nm and pixel dwell time of 1.20 µm. The acquisition of each time-series takes in total 26 seconds. The GFP fluorescence is excited with a white light laser source setting the wavelength at 489 nm and a detection window at 500–600 nm. The Images are recorded through a Hybrid photon counting detector in photon counting mode.

#### Data analysis

The acquired movies were analyzed using custom-written routines in Matlab to generate a map of the molecular brightness of the fluorescently labelled complexes (Fig. 2c) by calculating $${\epsilon }_{{\rm{ij}}}=({\rm{var}}({{\rm{I}}}_{{\rm{ij}}}(t))-{\rm{mean}}$$$$({{\rm{I}}}_{{\rm{ij}}}({\rm{t}})))/\mathrm{mean}({{\rm{I}}}_{{\rm{ij}}}({\rm{t}}))$$, for every pixel $$(i,j)$$. To minimize the effect of cell movement we use boxcar analysis^43^, with a boxcar filter size equal to 50 frames. In order to identify pixels belonging to the cell cytoplasm from background pixels, we then build a 2D histogram in which we group pixels that have similar values of mean intensity and brightness. With an elliptical tool, it is possible to select a region of the histogram and interactively check the corresponding region within the cell. This method helps avoiding pixels with brightness outliers and border regions that usually contribute with some extra-variance due to cell movement. Note that during the selection process from the histogram the brightness values are kept hidden to minimize any bias due to manual selection. The area of the histogram selected is finally fitted with a 2D Gaussian: $$f(I,{\rm{\varepsilon }})=a\,\exp \,[{(I-{I}_{0})}^{2}/2{s}_{I}+\,{({\rm{\varepsilon }}-{{\rm{\varepsilon }}}_{0})}^{2}/2{s}_{{\rm{\varepsilon }}}]$$ from the fit the parameters $${I}_{0}$$ and $${{\rm{\varepsilon }}}_{0}$$ give the mean intensity and brightness of the region of interest.

### Limited proteolysis

For limited proteolysis on cell lysates, cells were washed twice with ice-cold TBS (150 mM NaCl, 20 mM Tris-HCl, pH 7.5) and lysed in lysis buffer (100 mM KCl, 1 mM DTT, 0.5% Triton X-100, 25 mM HEPES-KOH, pH 7.5). The insoluble material was removed by centrifugation and protein concentration determined by Bradford protein assay (Bio-Rad). For limited proteolysis on cell lysates and on purified proteins, trypsin was diluted in lysis buffer and in 100 mM KCl, 25 mM HEPES-KOH, pH 7.5, respectively. Aliquots of lysates (50 μg protein) or purified proteins were incubated for 5 minutes on ice with different concentrations of trypsin. Proteolysis was stopped by denaturing the samples at 96°C, and samples analyzed by SDS-PAGE followed by immunoblotting with the indicated Abs (cell lysates). When indicated, filters for immunoblotting were subjected to acid stripping and re-probed with different antibodies.

### Production 6xHis-MBP-ERC1 and electron microscopy

Full length ERC1 obtained by PCR from GFP-ERC1 was inserted into a modified pOEM vector to produce His6-MBP-ERC1 for electron microscopy analysis.

#### Spodoptera frugiperda

Sf9 cells in ESF921 medium (Expression Systems) were co-transfected with linearized viral genome and the expression plasmid and selected for high infectivity. Viruses were produced and used to infect Sf9 cells and to obtain lysates for protein purification as described^44,45^. The 6xHis-MBP-ERC1 fusion protein was purified as previously described for extended coiled-coils in 20 mM HEPES pH7.4, 250 mM NaCl, 0.5 mM TCEP (46). Briefly, amylose resin was used to affinity isolate the dimeric ERC1 protein, subsequently eluted with 10 mM maltose, and subjected to size-exclusion chromatography. Protein concentration was determined by UV_280_ and Bradford assay.

The light-scattering from purified ERC1 was analyzed by an autosampler equipped Viskotek TDAMax system as described^45^. The data obtained were averaged across the protein elution volume and molecular masses determined by OmniSEC software package.

Samples for rotary shadowing were prepared as described^45^. Briefly, samples diluted in spraying buffer (100 mM ammonium acetate, 30% glycerol) were sprayed via a capillary onto freshly cleaved mica chips, which were then mounted in a high vacuum evaporator (MED 020, Baltec) and dried. Specimens were platinum coated (5–7.5 nm) and carbon was evaporated. Replicas were examined and imaged onto a CCD (Morgagni 268D, FEI; Morada G2, Olympus).

### Wound healing assays

MDA-MB-231 cells transfected for 24 h with GFP–tagged constructs, were re-plated in 96 well plates (40,000 cells in 100 µl of complete medium per well; 96-well ImageLock Plate, Essen BioScience, Ann Arbor, MI) and left to adhere for 3.5 h. 700 µm wide wounds were created with a WoundMaker Tool (Essen BioScience). Cells were washed with PBS, supplied with complete medium, and imaged every hour for 24 h with IncuCyte Live-Cell Imaging System equipped with 10x lens (Essen BioScience). For quantitative analysis, at each time point the number of GFP–positive cells in the wound within a selected field was expressed as percentage of total GFP–positive cells in the same field. Quantification was performed with the IncuCyte Scratch Wound Assays Software (Essen BioScience).

### Statistical analysis

Mean values are expressed ± SEM. Significant differences were evaluated by Student t-test, χ^2^ test, or non-parametric one-way ANOVA (*p < 0.05; **p < 0.01; ***p < 0.001). The colocalization of proteins at condensates (Figs 4d and 5b,c) was quantified as Pearson’s correlation coefficient using the Colocalization Finder plugin of ImageJ. For the analysis 19–35 areas with GFP-ERC1 condensates from 6–18 cells per experimental conditions were randomly picked from confocal microscopy images.

## Supplementary information

Supplementary information

Supplementary movie 1

Supplementary movie 2

Supplementary movie 3

Supplementary movie 4

Supplementary movie 5

Supplementary movie 6

Supplementary movie 7

Supplementary movie 8

Supplementary movie 9
