# Supplementary material for: The ERC1 scaffold protein implicated in cell motility drives the assembly of a liquid phase
Source: Sci Rep. 2019 Sep 19;9:13530. doi: 10.1038/s41598-019-49630-y (PMC6753080; doi:10.1038/s41598-019-49630-y)
Supplement: Supplementary file 1 — Supplementary information [file 41598_2019_49630_MOESM1_ESM.pdf]

Supplementary information

The ERC1 scaffold protein implicated in cell motility drives the assembly of a liquid phase

Kristyna Sala, Agnese Corbetta, Claudia Minici, Diletta Tonoli, David H. Murray, Eugenia Cammarota, Lucrezia Ribolla, Martina Ramella, Riccardo Fesce, Davide Mazza, Massimo Degano, Ivan de Curtis

Supplementary Figure Legends

Supplementary Figure 1. **ERC1-positive PMAPs in different cell types.** (**a**) Confocal frames from time lapse (**Supplementary movie 1**) of an MDA-MB-231 cell co-expressing the membrane marker GPI-GFP (green) and mCherry-ERC1 (red). (**b**) Confocal microscopy showing colocalization of endogenous ERC1 (blue), liprin-α1 (green) and LL5 (red) near the leading edge (arrows) of a HT1080 human fibrosarcoma cell. On the right, 2.5-fold enlargement of the area indicated by the asterisk in the left image. (**c**) Confocal microscopy of COS7 cells stained for the indicated endogenous proteins. Top panels: localization of endogenous liprin-α1 (green) near/at paxillin-positive (blue) focal adhesions. Bottom panels: arrows show examples of the colocalization of endogenous ERC1 (blue), liprin-α1 (green) and LL5 (red) near/at focal adhesions. (**d**) COS7 cells expressing GFP-ERC1 (green) stained with Nile Red (red). Scale bars, 20 µm.

Supplementary Figure 2. **Characterization of ERC1.** Related to **Figure 2**. (**a**) Top: probability to form two-stranded coiled coils as predicted by the program COILS (window 28). Below: the top scheme shows regions with high probability to form coiled coils, and a predicted C-terminal FIP-RBD domain; bottom scheme shows predicted IDRs as determined by DisEMBL. Disorder prediction in N-terminal ERC1 was confirmed by several other programs (not shown), including Iupred and Espritz, and by the D^2P^2 meta-analysis database. (**b-d**) N-terminal ERC1 is positively charged and enriched in disorder-promoting residues. (**b**) Sequence of murine ERC1a: positively charged residues Arg (R) and Lys (K) are in grey, negatively charged residues Asp (D) and Glu (E) in yellow. Positively charged N-terminal 147 residues are underlined; asterisks highlight a low complexity region (LCR, residues 34–51). (**c**) Percentage of order-promoting and disorder-promoting residues in the N-terminal ERC1(1-147) sequence, and in a set of intrinsically disordered proteins (IDPs) and structured (globular) proteins (***Tompa et al., 2002***). (**d**) Percentage of disorder-promoting (dark grey), order-promoting (light grey), and other residues (white) in ERC1(1-147) and full length ERC1. Bold numbers in ERC(1-147) column indicate positive or negative variations of at least two-fold with respect to the full length protein.

Supplementary Figure 3. Immunoblotting with anti-ERC1 after trypsinization. Related to **Figure 2**. Top: filters stained with Ponceau after blotting of gels loaded with lysates (50 µg

protein/lane) from cells transfected with the indicated ERC1 constructs, and incubated for 5 min at 0°C with increasing concentrations of trypsin. Bottom: two of the same filters shown in **Figure 2c** (left and central top filters**)** were reprobed with an Ab recognizing the N–terminus of ERC1, to highlight N-terminal ERC1 fragments.

Supplementary Figure 4. Characterization of the effects of GFP-ERC1 constructs on the formation of condensates. Related to **Figure 3.** Cells expressing the indicated GFP-tagged proteins were evaluated by fluorescence microscopy for: (**a**) efficiency of transfection, detected by fluorescence microscopy (n=102-165 cells); (**b**) total area of condensates per cell (n=35-42 cells); (**c**) mean area per condensate (n=934-1640 condensates). No significant difference could be detected between the three constructs. The increase observed for ERC1-Δ147 was not significant, due to the very large variability of the size of the condensates.

Supplementary Figure 5. Cytoplasmic condensates in the cytoplasm of cells expressing wildtype or truncated GFP-ERC1 constructs. Related to **Figure 3.** Confocal microscopy of cytoplasmic condensates in COS7 cells transfected with the indicated ERC1 constructs. Scale bar, 20 μm.

Supplementary Figure 6. Recruitment of client proteins at ERC1–induced condensates. Related to **Figure 5**. (**a**) Droplets positive for the indicated endogenous proteins in COS7 cells with GFP-ERC1–positive condensates (n=145–374); ***p<0.001 (χ^2 test to endogenous GAPDH). (**b**) COS7 cells co-expressing either GFP-ERC1 or GFP-ERC1-N together with FLAG-tagged constructs were used to quantify the percentage of either GFP-ERC1 droplets (n=229-409) or GFP-ERC1-N droplets (n=382-782) positive for liprin-α1 constructs; β-galactosidase, negative control. ***$p < 0.001$ by the χ^2 test; control, droplets in cells cotransfected with ERC1 construct and βgalactosidase.

Supplementary Figure 7. Endogenous liprin-α1 and LL5 proteins are not required to form ERC1 condensates. (**a**) COS7 cells coexpressing GFP-tagged ERC1 constructs with siRNAs for either liprin (siLip) or control siRNA (siLuc). Scale bar, 20 μm. Bottom: percentage of transfected cells with ERC1–positive droplets. *p<0.05 (n=3-4 experiments, 330-485 transfected cells). (**b**) COS7 cells coexpressing GFP-ERC1 with siRNAs for either LL5α and β proteins (siLL5s) or control siRNA (siLuc). Scale bar, 20 μm. Right: percentage

of transfected cells with ERC1–positive droplets (n=3 experiments, 545-695 transfected cells).

Supplementary movies - Legends

Supplementary movie 1. Confocal imaging of MDA-MB-231 cell co-expressing GPI-GFP and mCherry-ERC1; 65 min, 1 frame per min.

Supplementary movie 2. FRAP (circle) on migrating MDA-MB-231 cell expressing GFP-ERC1. 130 s, 1 frame every 2.7 s.

Supplementary movie 3. COS7 cells transfected with GFP-ERC1 imaged for 18 h, one frame every 5 min.

Supplementary movie 4. Fusion between two ERC1 droplets in a COS7 cell imaged for 42 s (one frame every 0.52 s).

Supplementary movie 5. Fission of an ERC1 droplet in COS7 cell imaged for 31 s (one frame every 0.52 s).

Supplementary movie 6. Photobleaching on a cytoplasmic ERC1–positive droplet (circle); 77 s, one frame every 0.52

Supplementary movie 7. Recovery of fluorescence after 2 μm diameter spot bleaching (arrow) on COS7 cell transfected with GFP-ERC1; 24 s, one frame every 0.2 s.

Supplementary movie 8. Recovery of fluorescence after 2 μm diameter spot bleaching (arrow) on COS7 cell transfected with GFP-ERC1Δ51; 24 s, one frame every 0.2 s.

Supplementary movie 9. Recovery of fluorescence after 2 μm diameter spot bleaching (arrow) on COS7 cell transfected with GFP-ERC1Δ147; 24 s, one frame every 0.2 s.

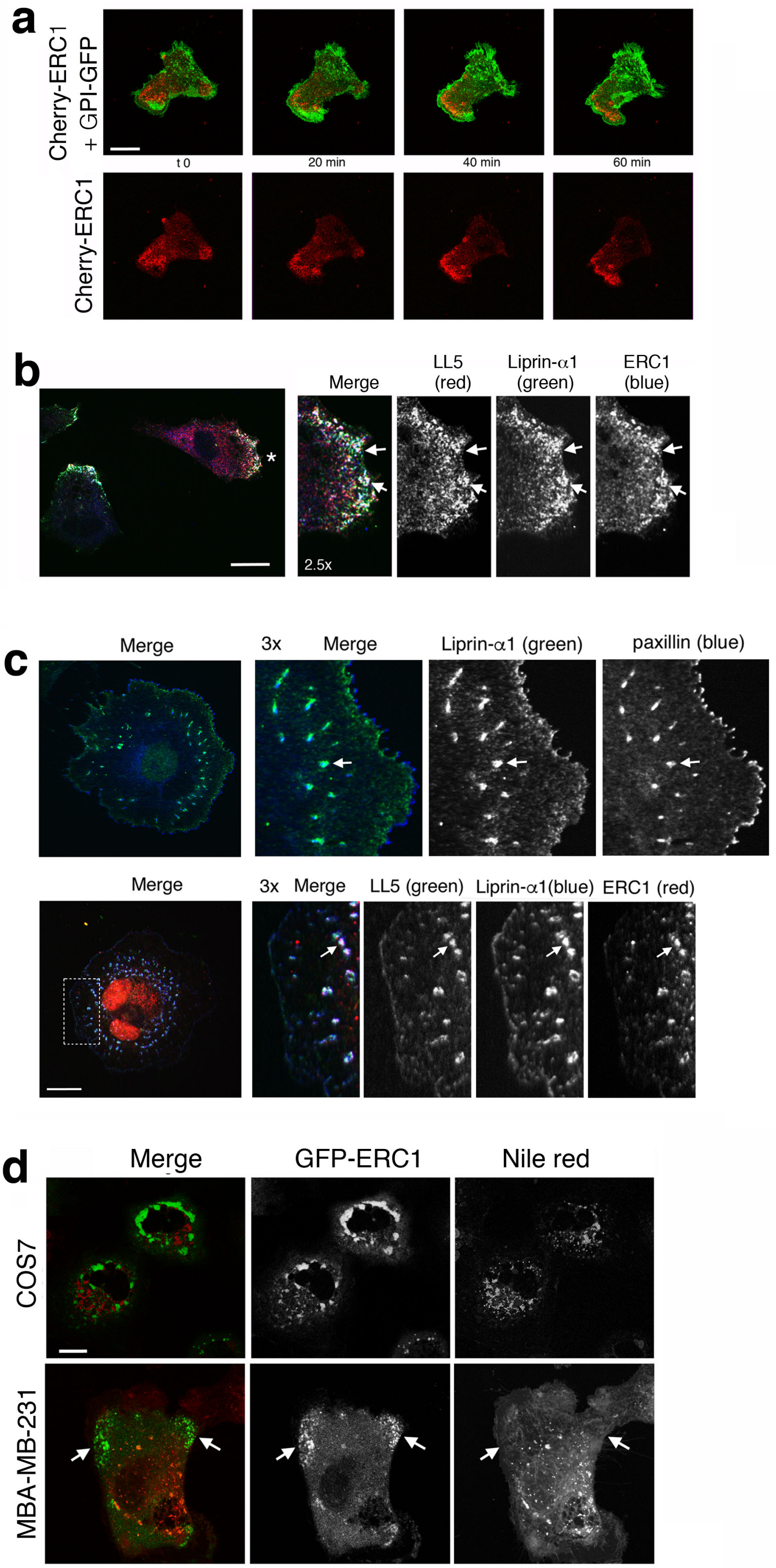

Supplementary Figure 1

a

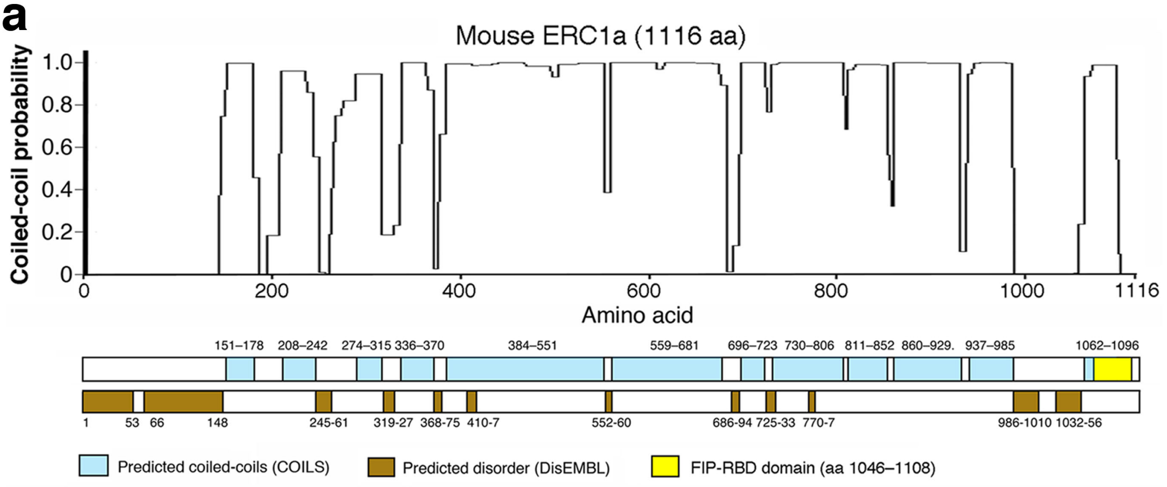

b

```
                  ***********************************************
MYGSARSVGKVEPSSQSPGRSPRLPRSPRLGHRRTNSTGGSSGNSVGGGSGKTLSMENIQ   60
SLNAAYATSGPMYLSDHENVGAETPRSTMTLGRSGGRLPYGVRMTAMGSSPNIASSGVAS  120
DTIAFGEHHLPPVSMASTVPHSLRQARDNTIMDLQTQLKEVLRENDLLRKDVEVKESKLS  180
SSMNSIKTFWSPELKKERALRKDEASKITIWKEQYRVVQEENQHMQMTIQALQDELRIQR  240
DLNQLFQQDSSSRTGEPCVAELTEENFQRLHAEHERQAKELFLLRKTLEEMELRIETQKQ  300
TLNARDESIKKLLEMLQSKGLSAKATEEDHERTRRLAEAEMHVHHLESLLEQKEKENNML  360
REEMHRRFENAPDSAKTKALQTVIEMKDSKISSMERGLRDLEEEIQMLKSNGALSSEERE  420
EEMKQMEVYRSHSKFMKNKVEQLKEELSSKDAQGEELKKRAAGLQSEIGQVKQELSRKDT  480
ELLALQTKLETLTNQFSDSKQHIEVLKESLTAKEQRAAILQTEVDALRLRLEEKETMLNK  540
KTKQIQDMAEEKGTQAGEIHDLKDMLDVKERKVNVLQKKIENLQEQLRDKEKQMSSLKER  600
VKSLQADTTNTDTALTTLEEALADKERTIERLKEQRDRDEREKQEEIDTYKKDLKDLREK  660
VSLLQGDLSEKEASLLDIKEHASSLASSGLKKDSRLKTLEIALEQKKEECLKMESQLKKA  720
HEATLEARASPEMSDRIQQLEREISRYKDESSKAQTEVDRLLEILKEVENEKNDKDKKIA  780
ELERQVKDQNKKVANLKHKEQVEKKKSAQMLEEARRREDSLSDSSQQLQDSLRKKDDRIE  840
ELEEALRESVQITAEREMVLAQEESARTNAEKQVEELLMAMEKVKQELESMKAKLSSTQQ  900
SLAEKETHLTNLRAERRKHLEEVLEMKQEALLAAISEKDANIALLELSSSKKKTQEEVAA  960
LKREKDRLVQQLKQQTQNRMKLMADNYEDDHFRSSRSNQTNHKPSPDQIIQPLLELDQNR 1020
SKLKLYIGHLTALCHDRDPLILRGLTPPASYNADGEQAAWENELQKMTQEQLQNELEKVE 1080
GDNAELQEFANTILQQIADHCPDILEQVVNALEESS                         1116
```

c

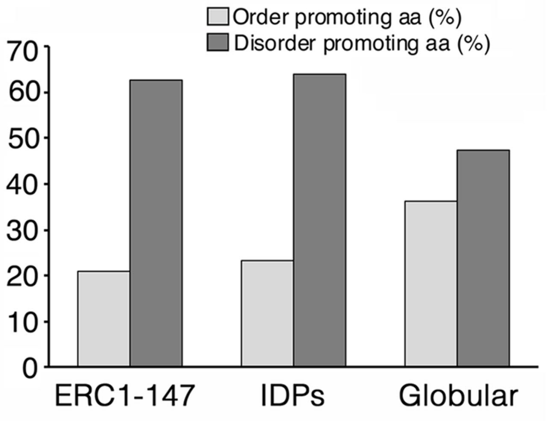

d

Amino acid	*ERC1-147*	*ERC1*
Ala (A)	7.5 %	7.1 %
Arg (R)	8.2 %	6.7 %
Gly (G)	**13.6 %**	3.1 %
Gln (Q)	**2.0 %**	7.4 %
Glu (E)	**3.4 %**	13.7 %
Lys (K)	**2.0 %**	9.9 %
Pro (P)	**8.2 %**	2.1 %
Ser (S)	**17.7 %**	8.7 %
Asp (D)	**1.4 %**	5.3 %
His (H)	3.4 %	2.5
Met (M)	4.8 %	3.3
Thr (T)	6.8 %	4.9
Asn (N)	4.1 %	3.7 %
Cys (C)	–	0.4 %
Ile (I)	2.0 %	3.4 %
Leu (L)	**6.1 %**	12.0 %
Phe (F)	0.7 %	0.9 %
Trp (W)	–	0.3 %
Tyr (Y)	**2.7 %**	1.0 %
Val (V)	21.0 %	25.6 %
Disorer promoting	62.6 %	58.7
Order promoting	21.0 %	25.6

Supplementary Figure 2

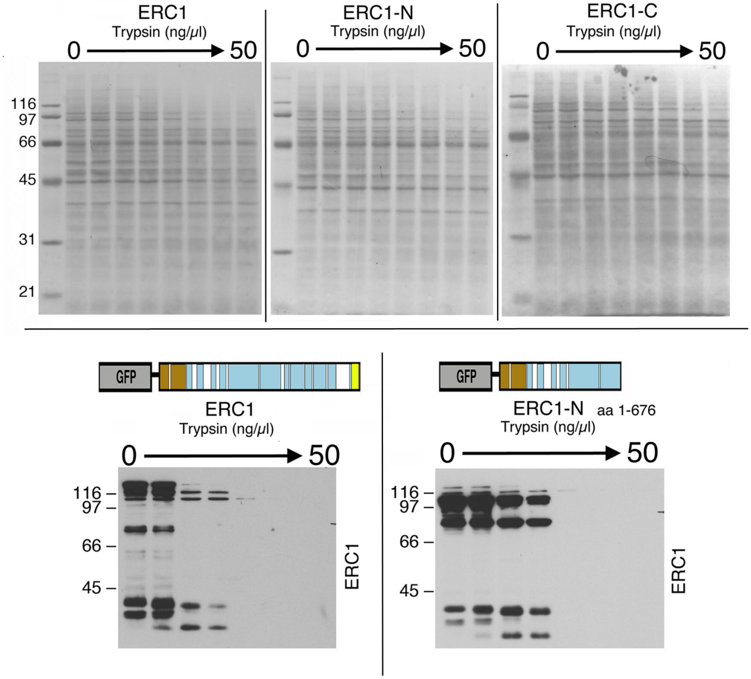

Supplementary Figure 3

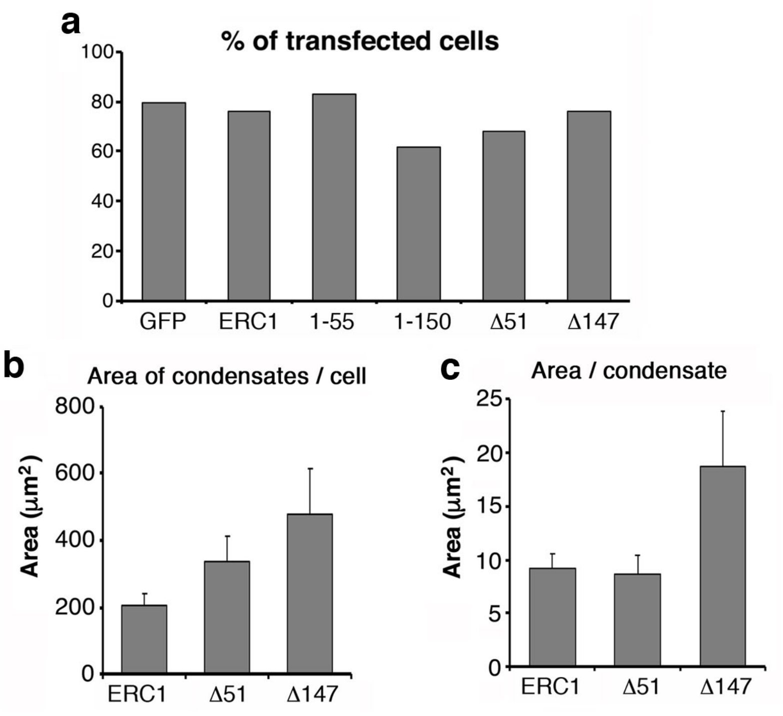

Supplementary Figure 4

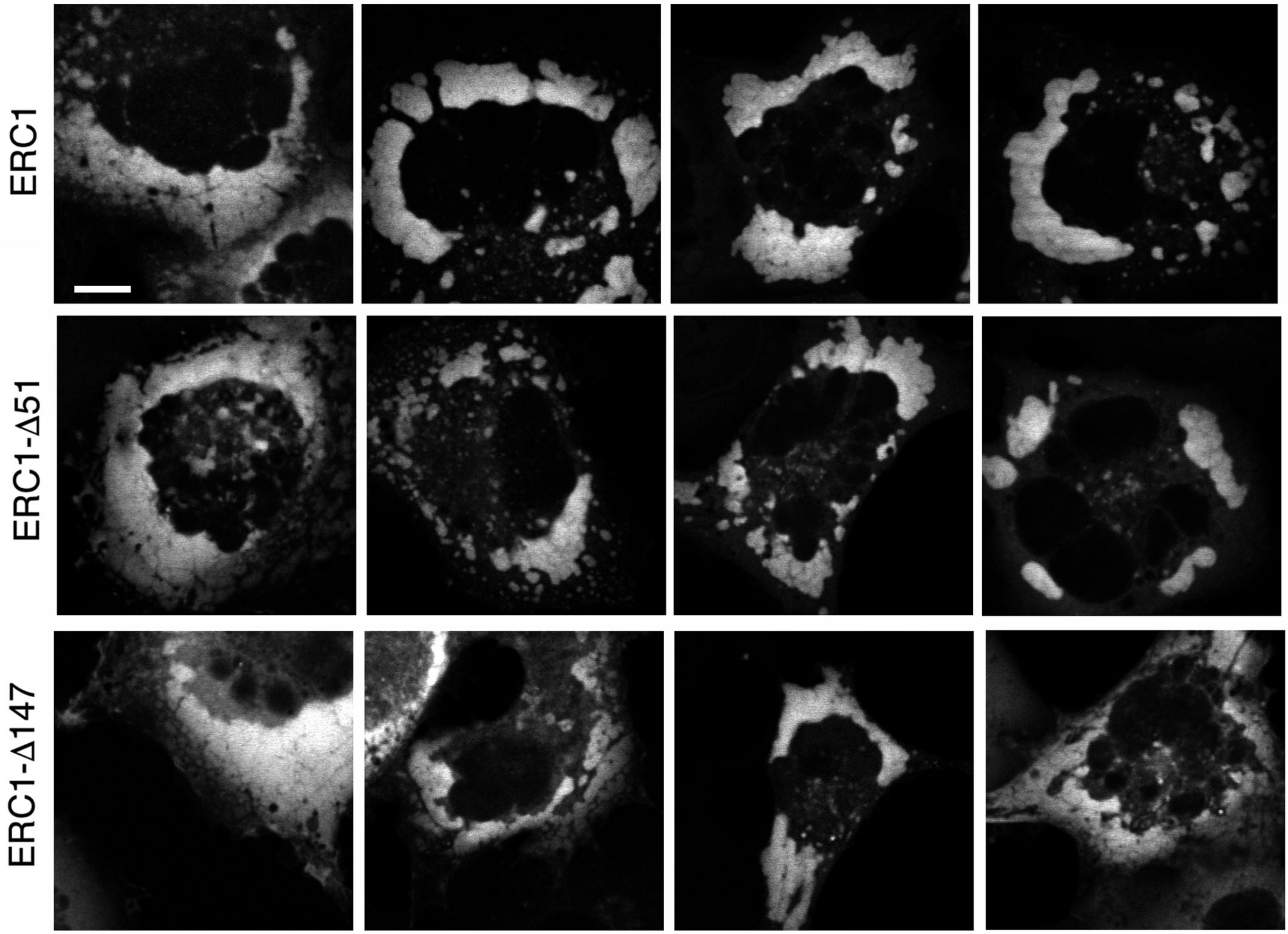

Supplementary Figure 5

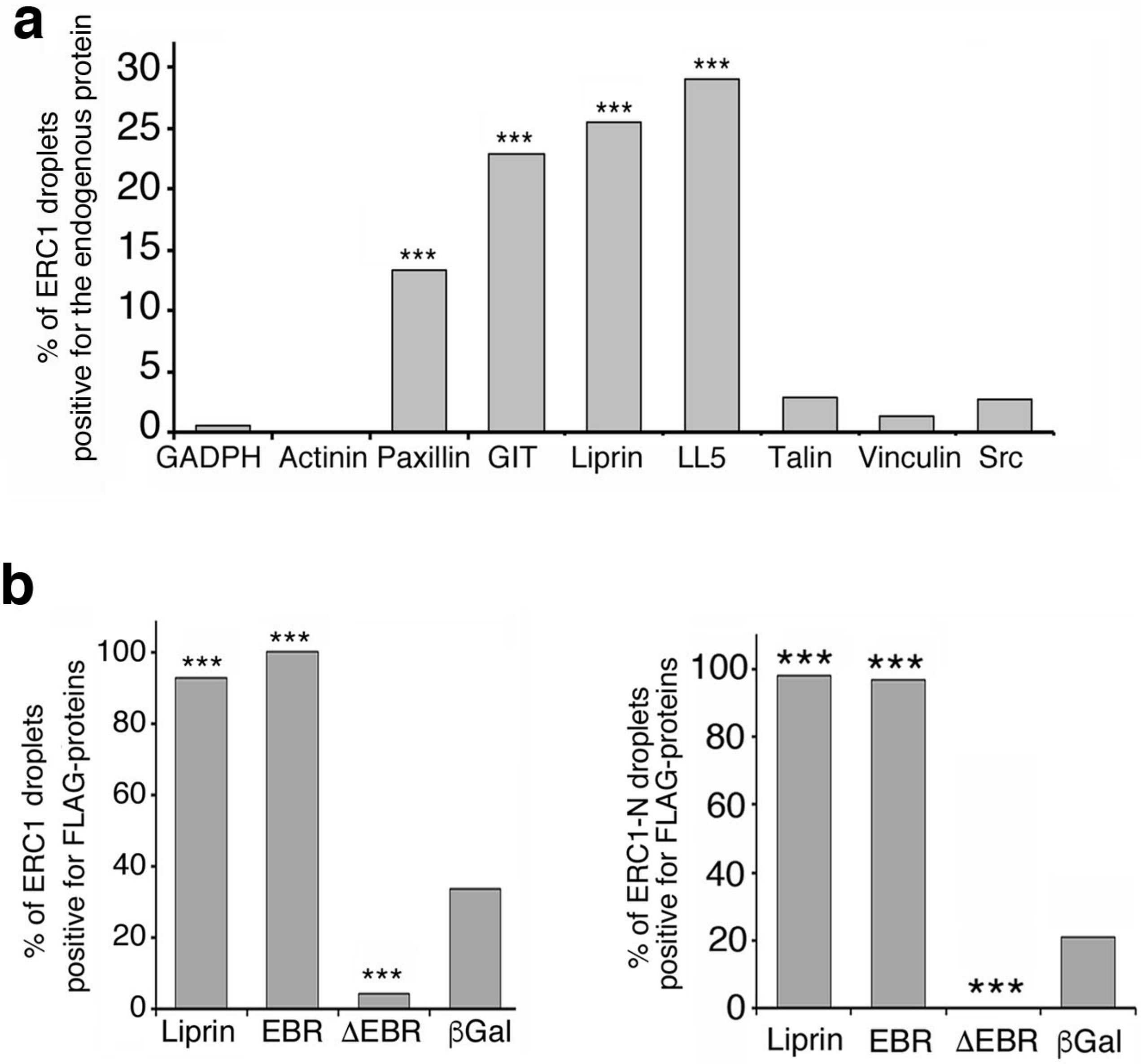

Supplementary Figure 6

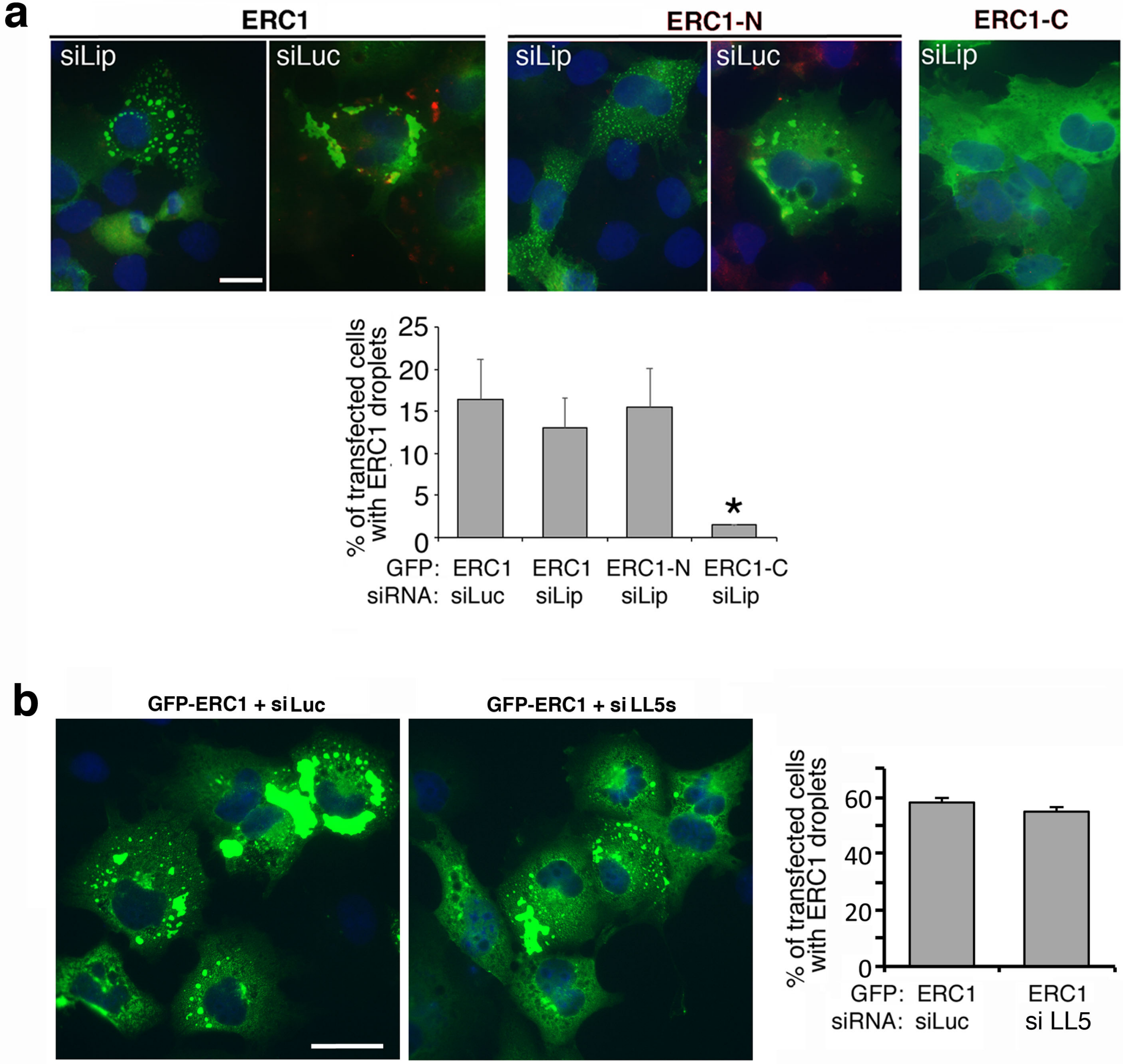

Supplementary Figure 7
